# How an international research funder’s forum developed guiding principles to ensure value and reduce waste in research

**DOI:** 10.12688/f1000research.128797.2

**Published:** 2023-12-28

**Authors:** Matthew Westmore, Michael Bowdery, Anne Cody, Kelly Dunham, Dorota Goble, Barbara van der Linden, Evelyn Whitlock, Elaine Williams, Cristina Lujan Barroso

**Affiliations:** 1 Health Research Authority, London, E20 1JQ, UK; 2 Health Care Research Wales, Cardiff, CF11 9AB, UK; 3 Health Research Board, Dublin, D02 H638, Ireland; 4 Patient-Centered Outcomes Research Institute, Washington, DC, 20036, USA; 5 National Institute for Health and Care Research, UK, Chilworth, SO16 7NS, UK; 6 ZonMw, The Hague, 2509 AE, The Netherlands; 7 School of Healthcare Enterprise & Innovation, University of Southampton, Southampton, SO17 1BJ, UK

**Keywords:** research funding, funding agency, funding policy, transparency, waste, value in research, international forum

## Abstract

**Background:** When health-related research funding agencies choose to fund research, they balance a number of competing issues: costs, stakeholder views and potential benefits. The REWARD Alliance, and the related Lancet-REWARD Campaign, question whether those decisions are yielding all the value they could.

**Methods:** A group of health-related research funding agencies, organisations that represent health-related research funding agencies and those that inform and set health-related-research funding policy from around the world have come together since 2016 to share, learn, collaborate and influence emerging practice. This group meets under the name of the Ensuring Value in Research Funders’ Forum (EViR Funders’ Forum). The EViR Funders’ Forum worked together to develop a set of ten Guiding Principles, that if funders adhered to would reduce research waste and ensure value in research.

**Results:** The EViR Funders’ Forum has previously agreed and published a Consensus Statement. The Forum has agreed on a set of ten Guiding Principles to help health-research funders to maximise the value of research by ensuring that: research priorities are justifiable; the design, conduct and analysis of research minimise bias; regulation and management are proportionate to risks; methods and findings are accessible in full; and findings are appropriately and effectively disseminated and used.

**Conclusions:** When setting research funding policy, we must balance multiple stakeholders’ needs and expectations. When funders do this well, they maximise the probability of benefits to society from the research they support - when funders do this badly, they passively allow or actively contribute to research waste. These challenges must be resolved by funders either working together or in conjunction with other actors in the research ecosystem.

## Introduction

The impact of health-related research, and the difference research makes for patients and the public, is the main focus of research funding agencies. It is the reason health-related research maintains the support of our health, public health and social care services and society more generally. But it is also essential to consider and acknowledge *how* funders deliver research systems and whether there are opportunities to enhance the efficiency and effectiveness of funding organisations.

Research funding agencies’ (RFAs) practice improvements are often set in the context of current global debates, specifically focusing on the purpose, accountability, and quality of research. These include issues raised about research integrity (World Conference on Research Integrity, https://wcrif.org/); the “crisis” of reproducibility in research ( Ioannidis, 2016); avoidable waste across the research enterprise ( Chalmers and Glasziou, 2009, Lancet Series Research: increasing value, reducing waste ( https://www.thelancet.com/series/research)); the impact, openness and transparency of specific health-related research activities (AllTrials, *All Trials Campaign*, http://www.alltrials.net/, Chalmers, Glasziou and Godlee, 2013); and the rise (and threat) of populism vis-a-vis science (Grant, 2017). Mostly these are well-meaning attempts to improve science; in some cases, they are biased attempts to undermine public confidence (Ignore the public at your peril, Times Higher Education (THE), https://www.timeshighereducation.com/news/ignore-public-at-your-peril).

Health-related research funding is provided through a range of sources such as the commercial sector, public resources (e.g. taxation or government borrowing) and philanthropic funding agencies, whose source funding comes from charitable activity such as raising donations or through a charitable trust. RFAs have mandated (e.g. through legislation) as well as internally developed policies and procedures that guide how they decide what research to fund and how funded research should be conducted and communicated. Research funders operate within a specific context, what the World Health Organisation would describe as an “integrated health research system” ( Pang *et al*. 2003). Although RFAs operate within different integrated research systems, defined by geographic, political, scientific and societal contexts, funding organisations share similar challenges. Whilst research funders can influence the environment in which they operate, they are one part of the wider research ecosystem.

Over the last 20 years, there have been a growing number of initiatives to improve health-related research practices across the spectrum of activities required to support research development, conduct and communication. These initiatives are often related to specific sectors of the research system (e.g. regulators or journal editors) or specific issues in the research process. Examples include the James Lind Alliance ( https://www.jla.nihr.ac.uk/), public involvement in research identification and prioritisation, research integrity, evidence synthesis methodology such as Cochrane’s innovative methods development and evidence synthesis for pre-clinical research via SYRCLE.

The Research Waste and Rewarding Diligence Alliance (REWARD) is a significant initiative in the world-wide efforts for quality improvement in research. The REWARD Alliance came from an initial paper on avoidable research waste in research in 2009 ( Chalmers and Glasziou, 2009), which was later followed up with a series (Lancet Series Research: increasing value, reducing waste, ( https://www.thelancet.com/series/research)), detailing expert consensus recommendations for all sectors of the research ecosystem. Both the REWARD Alliance and the 2014 Lancet series recommended aligned and collective action by different stakeholder groups – one such group suggested was research funders. The formation of the Ensuring Value in Research (EViR) Funders’ Forum, whilst not formally part of the REWARD Alliance, was strongly influenced by the work of the REWARD Alliance.

This paper sets out ten Guiding Principles, and a conceptual model, developed by the EViR Funders’ Forum in an attempt to contextualise and address these issues for funders. It will be of interest to RFAs considering their own policies and practice and researchers seeking to understand funders’ perspectives on these issues.

## Methods

To address the challenges facing health-related research, a group of health-related research funders, organisations that represent health-related research funders and those that inform and set funding policy from around the world, have come together to discuss these challenges, to share learnings, and to explore the potential for collaboration. The group started planning the Forum in 2016; the first meeting was held in January 2017. The group meets under the name of the Ensuring Value in Research Funders’ Collaboration and Development Forum (EViR Funders’ Forum, for short) ( Chinnery *et al.* 2018).

The Forum’s focus is on health-related research for two reasons: firstly, the initial members are health-related research funders; secondly, the debates on research waste and integrity, whilst relevant to all areas of research, are more established and have greater resonance given the implications, risks and opportunities associated with health-related research.

The National Institute for Health Research (NIHR) in the UK, the Patient-Centered Outcomes Research Institute (PCORI) in the US and the Netherlands Organisation for Health Research and Development (ZonMw) in the Netherlands planned and co-hosted the initial meetings. This steering group has grown since. Since its inception, the Forum has met twelve times, typically hosted by a different health-related funding organisation, or lately online due to travel restrictions:

1. London, England. NIHR – January 2017
2. The Hague, Netherlands. ZonMw – June 2017
3. Washington D.C., USA. PCORI – November 2017
4. Cardiff, Wales. Health Care Research Wales (HCRW) – May 2018
5. Canberra, Australia. National Health and Medical Research Council (NHMRC) – November 2018
6. Dublin, Ireland. Health Research Board (HRB) – March 2019
7. Washington, DC., USA. PCORI – September 2019
8. Berlin, Germany. REWARD-EQUATOR Conference 2020
9. Virtual – September 2020
10. Virtual – October 2021
11. Virtual – March 2022
12. Virtual – October 2022

**Figure 1.**
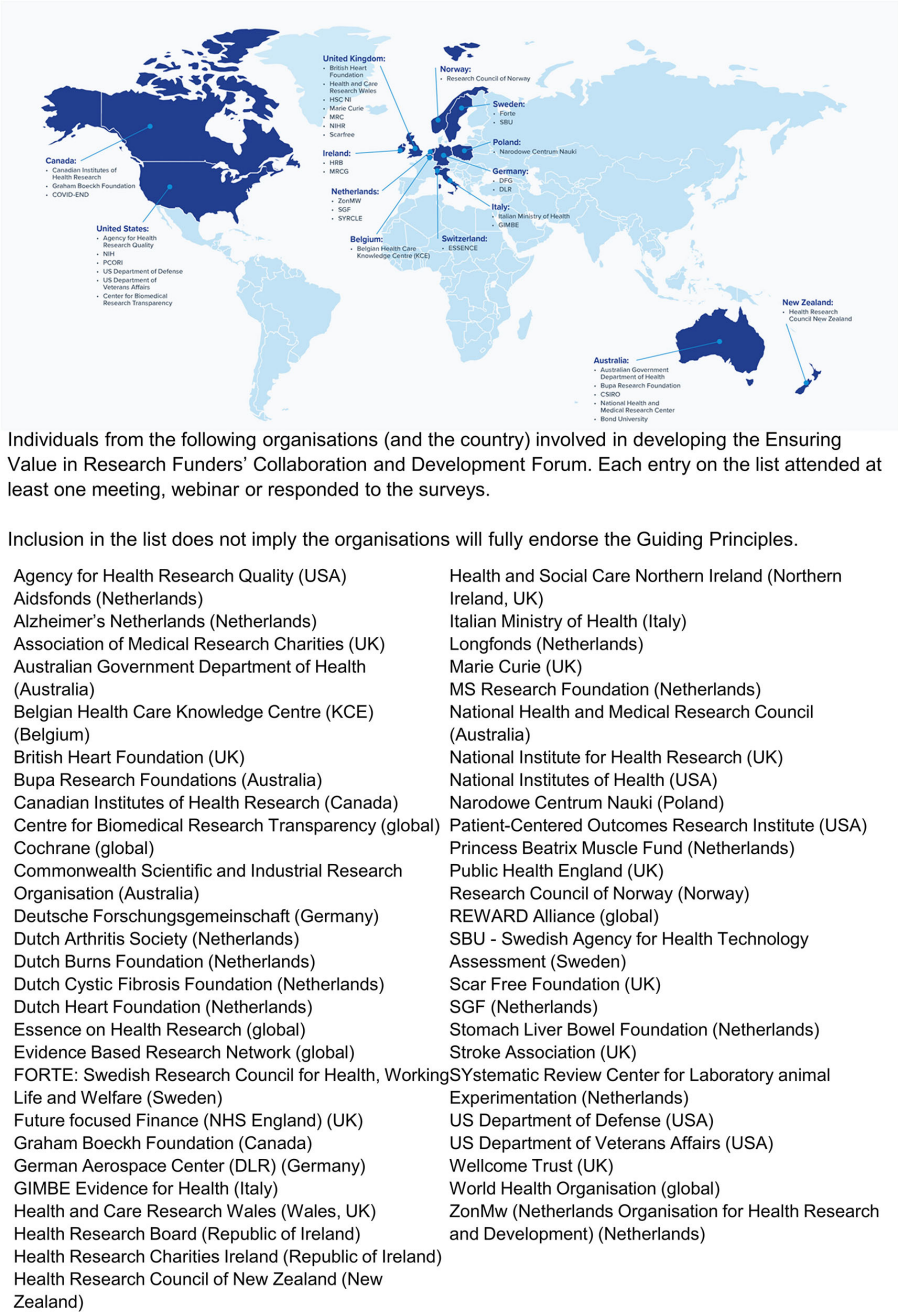
EViR engagement across the world.

Through meetings, webinars (added in 2018), and surveys of current practice, the EViR Funders’ Forum has interacted with 53 organisations that either fund health-related research, represent funders or are active contributors to this agenda. Figure 1 lists the organisations from whom at least one individual attended at least one meeting, webinar or responded to electronic surveys.

The Forum operates an open invitation policy whereby any public or philanthropic health-related research funding organisations, organisations that represent funders, or organisations that set health-related research funding policy, regardless of location, context, funding type, size or where on the research continuum (from basic discovery through to public health) may participate. The Forum also welcomes other non-funding related organisations to discuss the wider research system and specific topics of conversation. By encouraging diversity across a wide range of contexts, experiences and practices, the Forum provides a greater learning environment to start to address some of the uncertainties currently facing the whole research ecosystem. These practices also contribute to the broader purpose and aim of the EViR Funders’ Forum - to share experiences and to learn on what funders can do to maximise the probability of impact within and across respective research funding parameters.

### Initial activities

The initial activities of the EViR Funders’ Forum focused on developing a Consensus Statement and a set of Guiding Principles to serve as the backbone of our collaboration ( Chinnery *et al*. 2018). Surveys and sharing current practices through the surveys and open discussions were carried out to obtain and increase wider perspectives from other funding organisations.

## Results

**Figure 2.**
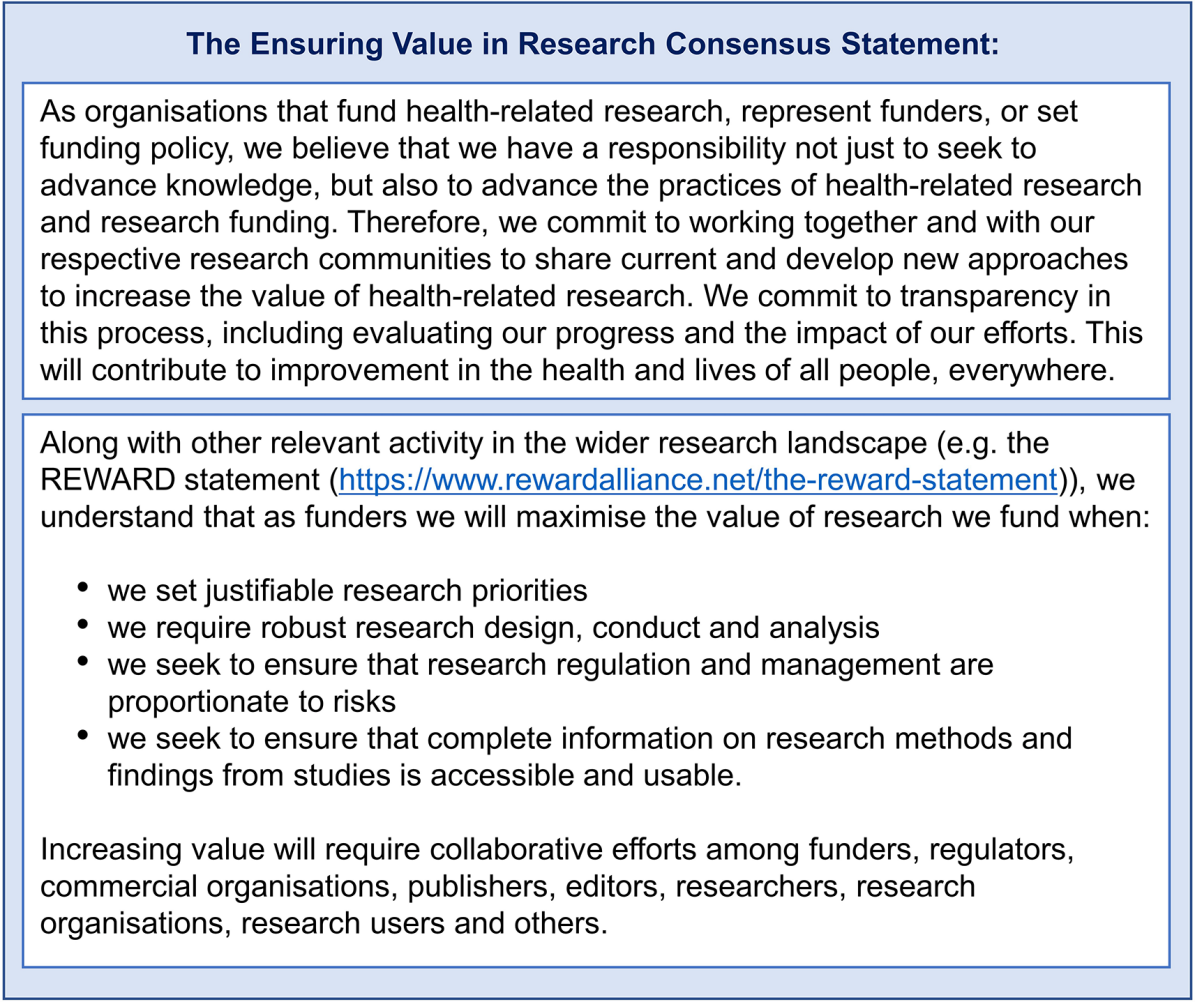
EViR Consensus Statement.

The development of the EViR Consensus Statement (see Figure 2) was an important step to ensure commitment, common purpose and shared understanding from funding organisations ( Chinnery *et al.* 2018).

Following the Consensus Statement, the Funders’ Forum began to develop the EViR Guiding Principles. These principles are an extension of the Consensus Statement to help guide funders to ensure their work delivers the greatest value. The NIHR’s Adding Value in Research Framework (AViR, https://www.nihr.ac.uk/about-us/our-contribution-to-research/how-we-are-improving-research/adding-value-in-research.htm) was used to help shape the Guiding Principles. The NIHRs AViR Framework was initiated in response to the research waste, research integrity and research transparency literature [ https://www.nihr.ac.uk/about-us/our-contribution-to-research/how-we-are-improving-research/adding-value-in-research.htm, and as a result of Lancet avoidable waste series (2014)]. The Guiding Principles were iterated and agreed by consensus during the first three EViR Funders’ Forum meetings. The ten Principles are published on the EViR website ( https://evir.org/our-principles/) and reproduced in Table 1.

**Table 1.** The Guiding Principles (EViR 2021).

*The Guiding Principles (EViR 2021)*	
*Setting justifiable research priorities*.	
**Principle 1**	Health-related research agendas and priorities should be set with the meaningful involvement of those who will use and be affected by health-related research.
*Ensuring robust research design, conduct and analysis*.	
**Principle 2**	Research should only be funded if set in the context of one or more existing systematic reviews of what is already known (or an otherwise robust demonstration of a research gap).
**Principle 3**	Funders should take into account advances in research methodology and fund new research only if adequate steps have been taken to reduce bias.
*Ensuring regulation and management of research conduct are proportionate to the costs and risks of research.*	
**Principle 4**	Selection and conduct of research should be actively managed in a risk proportionate way, consistent with applicable human participant research laws, regulations, and ethical guidance.
**Principle 5**	Studies should be registered in an appropriate, design-relevant publicly accessible registry at study inception whenever possible.
*Ensuring all information on research methods and findings are accessible and all reports are complete and usable*.	
**Principle 6**	Research questions, methods, materials, analysis plans or sequence of analytical choices for all studies should be made available as early as possible and preferably near or before the start of the study or analysis. Any deviation from the original plans should be documented.
**Principle 7**	All studies should report methods and findings in full, following credible and justifiable reporting guidelines. This applies irrespective of the nature of the findings and whether the study completed as planned.
**Principle 8**	When appropriate and when it will add value to evidence users, replication, re-analysis, and re-use of data from studies should be supported and facilitated.
**Principle 9**	New evidence should be placed in the context of existing knowledge to inform appropriate interpretation and use of findings. When appropriate and when it will add value to evidence users, systematic reviews should be updated following primary research.
**Principle 10**	Research knowledge that can lead to benefit should be effectively disseminated with and to end users. Where appropriate, the usage of new knowledge should be supported and facilitated.

### Tying it all together: A conceptual model

**Figure 3.**
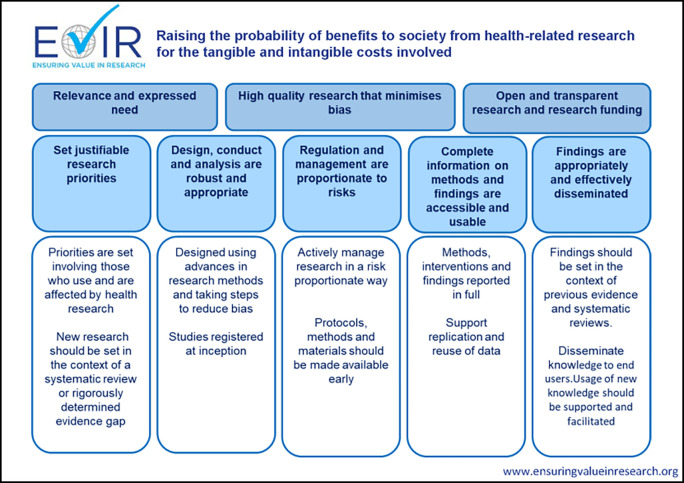
Summary of Funders’ Forum Guiding Principles and conceptual model.

Beyond statements of intent and guiding principles, funders must consider how well their policies and practices align and support their mission of funding research that is credible and beneficial to the public. The ten Guiding Principles provide the practical link between the conceptual domains in the Consensus Statement to the actual policies, procedures and activities delivered by funders. The resulting conceptual model underpinning work of the EViR Funders’ Forum is shown in Figure 3.

**Figure 4.**
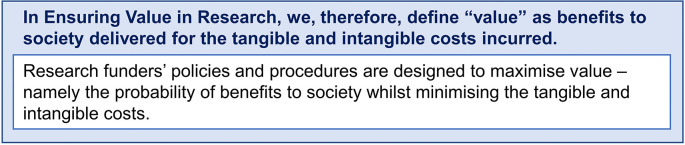
EViR “value” definition.

Many funders will describe the outcomes they are trying to achieve as impact. The EViR Funders’ Forum has deliberately not defined impact, as this is a much broader topic whose importance will be specific to an individual funder and context. However, the Forum does recognise that the probability of positive impact (benefit) should be enhanced through the application of these principles and practices designed to maximise value (see Figure 4). The model also draws on a tacit understanding that impacts are in relation to benefits accrued to society rather than impacts in relation to surrogate outcomes, such as academic publications and their related metrics. This would also include the impact research has on research itself; for example, by changing research agendas, opening up new avenues of exploration that could lead to further benefits, and closing down others that could not.

Funders are seeking to maximise the benefits their research delivers and ensure these are relative to the research costs incurred. Cost is interpreted broadly to include tangible costs such as time and money and intangible costs such as opportunity costs, political support, public support, participant and stakeholder experience and enthusiasm, patient and public participation.

To maximise the probability of benefit (impact) relative to the tangible and intangible costs incurred, funders’ policies and procedures should aim to ensure the research they support is:

- Relevant to the intended end user of the research (e.g. patients and clinicians for clinical research; the next research community in the translational pathway for earlier phase research).
- High quality and with minimal bias.
- Transparent and open. This should also apply to funders’ activities.

These high-level aims are achieved by ensuring that:

- Research priorities are justifiable.
- Design, conduct and analysis are robust and appropriate.
- Regulation and management are proportionate to risks.
- Complete information on research methods and findings are publicly accessible and in usable formats.
- Findings are appropriately and effectively disseminated.

An integrated conceptual model, as outlined in Figure 1, allows funding organisations to think coherently and comprehensively about their research practices to identify key areas for improvement. Policies and practices can be investigated and evaluated to determine their alignment with all or some of the Guiding Principles outlined in this paper. Conducting performance audits of specific policies can then help to guide quality improvement across a range of funders and within different contexts (e.g. Boutron *et al.* 2016, Nasser *et al.* 2017, Whitlock *et al.* 2018, Cody *et al.* 2021). The Forum is currently piloting an audit tool for members to track their performance against the conceptual model and Guiding Principles.

## Discussion

For funders of health-related research, this work provides a logic model of the indicators of a high value research funder. The Guiding Principles are an aspiration we work to, rather than a checklist or a target to be achieved. As research policy and practice evolve, the interpretation of each Principle will accrue additional facets. For example, what constitutes a robust research design will change over time as new methods become available, and the ways in which research protocols and findings can be shared continue to increase. As such, the Guiding Principles give structure and focus to continual improvement strategies, first with policies and procedures and then with monitoring and measurement.

Despite the increased attention since the original paper on avoidable waste in research, Chalmers and Glasziou lamented that “research waste is still a scandal” but also recognised that the establishment of the EViR Funders’ Forum is “… perhaps the most notable and potentially influential development …” in addressing avoidable waste in research ( Glasziou and Chalmers, 2018).

### Implications

The Forum has used the Guiding Principles to share their experience, learn from each other and collaborate to move its collective understanding forward. Diverse topics have been discussed against the framework of the Guiding Principles: for example, data sharing, automation, priority setting, novel decision-making processes, and transparency.

Longer-term working groups have focussed on implications for preclinical research ( Ritskes-Hoitinga *et al.* 2018); stakeholder engagement; dissemination and implementation; systematic reviews ahead of new primary research; and self-audit against the Guiding Principles as a whole.

To date, funders in the Forum have used the Guiding Principles as a tool for internal measurement, process improvement, and strategy development. The Research Quality Committee of the National Health and Medical Research Council in Australia ( NHMRC 2019) used the Guiding Principles to inform the process of developing its recently released Research Quality Strategy, the aim of which is to ensure the highest quality and value of NHMRC-funded research. The Health Research Council of New Zealand has shared the Guiding Principles with its statutory Research Committees with responsibility to provide advice to Council on the assignment of funds, and shared information in New Zealand Health Research Strategy cross-agency discussions with a view to increasing awareness and enhancing engagement in ensuring value in research. The National Institute for Health and Care Research in the UK has fully incorporated all ten Guiding Principles into its own Adding Value in Research Framework and is developing new areas of work to deliver on them.

Funders in the Forum have also applied the Guiding Principles to standards/requirements for researchers seeking funding. The Scar Free Foundation now requires applicants for funding to embed the EViR Guiding Principles in the study design, management and dissemination of their research. Several funders now require researchers to demonstrate that there is a need for the proposed study that is informed by a systematic review or other robust evaluation of the available evidence (Italian Ministry of Health, NIHR, NHMRC, PCORI, HCRW, HRB and the Stroke Association). While NIHR and PCORI had this requirement prior to the formation of the Funders’ Forum, others have been prompted by their participation in the Forum.

One of the authors (MR-H) has led a linked project to highlight ways to apply the Guiding Principles to pre-clinical research by adding pertinent examples. In 2020, a pre-clinical working group was established which will focus on furthering the implementation of the Guiding Principles for pre-clinical research as well.

It is important that funders understand current practice internationally and evaluate progress in improving research systems. The Guiding Principles provide a framework for this discussion. Some principles lend themselves to simple quantitative measures (e.g. percentage of studies registered, Knowles 2020), while others are more complex and qualitative. Even those that may at first appear more straightforward need to be seen in context: for example, whilst regulated clinical trials are required by law to be registered, the same does not apply to other types of health research and practice varies between health services research, biomedical research, and population health research. Funders and the wider community will need to develop methods for monitoring and tracking these process measures. In doing so, it is important to remember that these will inevitably only be indicators. These indicators are useful because they are largely within our collective influence within medium term time frames. However, they should not be confused with the end aim; namely, maximising the benefits to society from research, which is the end state of a complex system.

There are also implications for research-on-research or meta-research. Research-on-research uses a range of research methods to investigate the process of research or of funders’ policies and guidance with the aim of improving the planning, conduct and sharing of research as well as its governance and oversite, including how funding decisions are made. The concepts in this paper are grounded in the research-on-research literature but it is fragmented and often descriptive. More research is required under each guiding principle. The Guiding Principles may provide the framework for a research-on-research agenda that can provide evidence regarding funders’ policies and practice. This is already beginning to happen; the NIHR has a long running research-on-research programme that has used the concepts and issues discussed here as an organising framework for its research agenda (NIHR research on research (RoR), https://www.southampton.ac.uk/netscc/research/index.page).

This work highlights how funders are placing greater emphasis on research practices and by doing so contribute to the improvements of the research they fund. The Funders’ Forum and its Guiding Principles will continue to translate into funder policies and procedures, and to ensure value in research.

## Conclusions

A research funder’s policies and procedures are aimed at ensuring the relevance, quality and transparency of the research process. When RFAs do this well, they will maximise the probability of benefits to society from the research they support - when they do this badly, they passively allow or actively contribute to research waste. This requires continuous, committed, concerted and collaborative effort. The funders within the EViR Funders’ Forum do just that – they work together to effect change within our own organisations and within the wider global research system.

We hope other organisations want to be involved in the Forum, so if you would like any further information about our work, or if your organisation is interested in joining the Forum, please contact evirfundersforum@gmail.com.

## Data Availability

Full agendas of the EViR Funders’ Forum meetings, redacted only to remove personal data, are available in the public domain on https://evir.org/events-and-outputs/events/. Due to data protection concerns, minutes of the meetings are available on request, please contact the EViR secretariat ( evirfundersforum@gmail.com) for more information.
