# Supplementary material for: How an international research funder’s forum developed guiding principles to ensure value and reduce waste in research
Source: F1000Res. 2023 Dec 28;12:310. Originally published 2023 Mar 21. [Version 2] doi: 10.12688/f1000research.128797.2 (PMC11153987; doi:10.12688/f1000research.128797.2)
Supplement: Peer review history

# Reviewer report: Reviewer response for version 2

**DOI:** 10.5256/f1000research.160448.r272999

**Published:** 2024-06-05

**Authors:** Maximilian Siebert

**Affiliations:** 1 Stanford University, Stanford, California, USA

**Summary**

 This manuscript provides a detailed account of the activities, principles, and objectives of the Ensuring Value in Health Research Funders' Forum. As a collaborative endeavor involving health-related research funders, the forum focuses on overcoming common obstacles in research funding, enhancing research methodologies and amplifying the value and impact of health research investments.

 The article delineates 10 guiding principles aimed at establishing justifiable priorities in health research, ensuring robust regulation and management and promoting accessibility to comprehensive information on research methods and outcomes.

**Importance and Originality**

 This article is both timely and vital, emphasizing the necessity of upholding research integrity to enhance the value delivered in health care and patient outcomes.

**Comments**

 As this manuscript is in its second round of reviews, I have just two minor suggestions.

 The Comments of Hans Lund have been addressed.

 1.

 It is recommended that the title explicitly reference its focus on health research to prevent potential misunderstandings among readers.

 2.

 The manuscript should include an expanded discussion on the geographic distribution of member institutions, all of which are situated in high-income countries. An analysis of how this may shape the formulated principles is crucial, especially considering that the predominant burden of disease lies in lower-income regions. This will provide a more comprehensive understanding of the forum’s global impact and relevance.

Is the work clearly and accurately presented and does it cite the current literature?

Yes

If applicable, is the statistical analysis and its interpretation appropriate?

Not applicable

Are all the source data underlying the results available to ensure full reproducibility?

No source data required

Is the study design appropriate and is the work technically sound?

Yes

Are the conclusions drawn adequately supported by the results?

Yes

Are sufficient details of methods and analysis provided to allow replication by others?

Yes

Reviewer Expertise:

Epidemiology, Meta Research

I confirm that I have read this submission and believe that I have an appropriate level of expertise to confirm that it is of an acceptable scientific standard.

# Reviewer report: Reviewer response for version 2

**DOI:** 10.5256/f1000research.160448.r233984

**Published:** 2024-02-28

**Authors:** Till Bruckner

**Affiliations:** 1 QUEST Center for Transforming Biomedical Research, Berlin Institute of Health (BIH), Charité - Universitätsmedizin Berlin, Berlin, Germany

I welcome the indexing of this paper and have no further comments to make.

Is the work clearly and accurately presented and does it cite the current literature?

Yes

If applicable, is the statistical analysis and its interpretation appropriate?

Not applicable

Are all the source data underlying the results available to ensure full reproducibility?

No source data required

Is the study design appropriate and is the work technically sound?

Yes

Are the conclusions drawn adequately supported by the results?

Yes

Are sufficient details of methods and analysis provided to allow replication by others?

Partly

Reviewer Expertise:

Clinical trial registration and reporting, meta-research, regulatory science

I confirm that I have read this submission and believe that I have an appropriate level of expertise to confirm that it is of an acceptable scientific standard.

# Reviewer report: Reviewer response for version 1

**DOI:** 10.5256/f1000research.141421.r168745

**Published:** 2023-04-19

**Authors:** Hans Lund

**Affiliations:** 1 Section Evidence-Based Practice, Department of Health and Functioning, Western Norway University of Applied Sciences, Bergen, Norway

The authors and the organisation behind should be praised for their initiative and for promoting a better and more transparent approach to funding health research.

 I suggest the authors to consider the following comments and questions:

1. Maybe explain a little more in details how figure 2, table 1, figure 3, and figure 4 were developed. How many participated in the process? What were the diversity of the group for example geographically, experience with funding or as applicant? How was consensus achieved? and alike.
2. The authors mentioned the importance of meta-research both as inspiration for the Consensus Statement, Guiding Principles, the conceptual model and their definition of value in research, and in the Discussion. As it can be very difficult to find funding calls including meta-research, and even big difficulties in finding funding support for meta-research in open calls, it would have been good if the need of research evaluating researchers and research practice, evaluating research ethic committees practice, funders practice and scientific journal practice was highlighted in the Guiding Principles.
3. The authors should maybe consider an alternative definition of value in research or valuable research (see Figure 4): For science to be of value it needs to be both scientific valid and relevant. Thus, valuable research could be defined as the product of scientific validity and the societal relevance of the study in relation to the costs (Valuable Research = Validity x Relevance / Costs). Validity deals with how research is designed and carried out to reduce bias, including whether research is answering a research gap. Relevance deals with the importance of the research for society in general and the needs of the intended end users specifically. In this context, end users can be defined as those who will either use the results of the research and/or be affected by the results. Costs in the equation includes the resources used in conducting the study and the risks study participants are taking.
4. On page 8 the authors refers to Figure 1, but maybe they should have referred to Figure 3?
5. One way to improve the use of means for health research is by carrying out different forms of research priority setting processed. The results from such processes can inform the researchers when arguing for a new study, but it could also be an important source of prioritisation of funding calls. The authors should consider to include this in the Discussion. A recent study indicates that such processes lacks both systematicity and transparency and may be of relevance for the discussion in the present paper. (See: Lund et al. 2022) ^1^.

The paper is a very important contribution to diminish waste in research, and by providing suggestions for how funding decisions could be made it is possible to have more relevant and important studies supported in the future. Thanks for this brilliant initiative.

Is the work clearly and accurately presented and does it cite the current literature?

Yes

If applicable, is the statistical analysis and its interpretation appropriate?

Not applicable

Are all the source data underlying the results available to ensure full reproducibility?

No source data required

Is the study design appropriate and is the work technically sound?

Yes

Are the conclusions drawn adequately supported by the results?

Yes

Are sufficient details of methods and analysis provided to allow replication by others?

Partly

Reviewer Expertise:

Evidence-Based approaches to clinical practice, to research (for example: evidence-based research) and to teaching. Meta-research.

I confirm that I have read this submission and believe that I have an appropriate level of expertise to confirm that it is of an acceptable scientific standard, however I have significant reservations, as outlined above.

## References

[1] : Lack of systematicity in research prioritisation processes - a scoping review of evidence syntheses. *Syst Rev* .2022;11(1) : 10.1186/s13643-022-02149-2 277 10.1186/s13643-022-02149-2 36564846 PMC9784020

# Response

**Published:** 2023-12-19

**Authors:** Cristina Lujan

**Affiliations:** University of Southampton, UK

Dear Professor Hans Lund,

 We would like to thank you for reviewing our paper and responding so positively to this work. We are particularly grateful for your insightful comments, which we fully agree with, even if some we were unable to amend the paper directly. Please find our responses below:

**Comment 1. **All artefacts were developed and approved through the forum meetings. Final versions were ratified by the steering group and presented back to the full list of individuals and organisations that attended the meetings. Whilst different members of the Forum joined at different times, and different members were active in the discussion to a greater or lesser extent, the diversity of input is reflected in Figure 1.

**Comment 2.** The authors thank the review for this insightful comment. It is noted and the authors agree. This point was discussed at a number of meetings but it did not become part of the agreed guiding principles and so it would not be appropriate to change the agreed principles to incorporate this point. However, in the recent EViR Strategic Workplan this has been acknowledged as a priority area ( https://evir.org/events-and-resources/resources/evir-strategic-workplan-expanding-our-vision/) and work is currently ongoing with experts in the field.  

**Comment 3.** This is an interesting view that clearly has merit. The definition of value we used was purposefully centred on real world impact or outcomes and was inspired by Michael Porter's definition of healthcare value in http://www.nejm.org/doi/full/10.1056/NEJMp1011024?viewType=Print&

 The reason for choosing a definition firmly related to real world impact was to ensure that our focus was on real world benefits rather than outputs or process issues like scientific validity and relevance. This allowed a diverse group of funders, from across the world, with different political, social and scientific contexts, from pre-clinical and early phases to health service research, to align under the idea that we are all interested in delivering benefits to society rather than just good science. As well as creating a sense of alliance, to be candid, this prevented us from discussing different views and definitions of research impact, and meant we could focus on the task at hand, which was to develop useful guidance to change funder practices.

 That said, the reviewer's points are entirely correct, which is why we structured our framework (figure 3) under the headings of relevance, quality (akin to validity), and openness. It could be interpreted that we were saying value = (relevance x quality x openness) / costs, which aligns with the reviewer's thinking. We did not articulate it that way in the agreed version, so while we don't disagree with the reviewer, it would not be appropriate to change the text now.

**Comment 4. **We agree, and the manuscript has been updated.

**Comment 5. **We agree with this point. Unfortunately, the journal prevented us from including further details on why each guiding principle is important and some practical examples of how to delivery them because we had already placed this material on our website. We can not add this to the paper but the reviewer may be reassured that we agree and  have included this on our website - Applying the principles - EViR

# Reviewer report: Reviewer response for version 1

**DOI:** 10.5256/f1000research.141421.r167144

**Published:** 2023-03-24

**Authors:** Till Bruckner

**Affiliations:** 1 QUEST Center for Transforming Biomedical Research, Berlin Institute of Health (BIH), Charité - Universitätsmedizin Berlin, Berlin, Germany

This paper provides a narrative overview of the creation and evolution of the EViR Funders’ Forum and its development of a Consensus Statement and Guiding Principles to help health research funders to maximise the value of research. It is a valuable addition to the literature because it describes an innovative platform that allows and enables diverse funders from different countries to jointly discuss how to address issues of common concern.

 One arguable shortcoming of the paper is that it fails to explore the more ‘political’ (in the broadest sense) aspects of funders’ engagement with the issues of research waste and research quality. Funders may face considerable barriers to acting decisively to maximise the value of research. For instance, openly acknowledging that funders may not be making optimal use of public money could invite criticism from political decision-makers and taxpayers and threaten their budgets. Another barrier is that funders often seem determined to maintain positive relationships – including long-standing personal relationships – with the research community, which disincentivises the imposition of sanctions.

 For example, the world’s largest medical research funder, the National Institutes of Health (NIH), has continued to award new funding to grantees that had violated NIH’s own policies and thereby generated research waste ( https://www.statnews.com/pharmalot/2022/10/14/clinical-trials-nih-transparency/). In general, there often seems to be a gap between funders’ publicly stated aspirations and their subsequent translation into practice. In this context, it is excellent that the authors cite Knowles et al., who focuses on the thorny issue of implementation.

 However, given space constraints, omission of these more ‘political’ aspects from the scope of the article is defensible, especially given that the Consensus Statement and Guiding Principles provide a useful starting point for further debate about funders’ upstream aspirations. The Forum’s experiences gained during the ongoing piloting of an audit tool might provide a good starting point for a future, separate publication that focuses on the downstream challenges of implementation.

 In sum, this paper is a valuable addition to the literature. I look forward to future publications documenting the Forum’s further development and experiences over time.

 Till Bruckner

 TranspariMED

 Bristol, UK, 23 March 2023

Is the work clearly and accurately presented and does it cite the current literature?

Yes

If applicable, is the statistical analysis and its interpretation appropriate?

Not applicable

Are all the source data underlying the results available to ensure full reproducibility?

No source data required

Is the study design appropriate and is the work technically sound?

Yes

Are the conclusions drawn adequately supported by the results?

Yes

Are sufficient details of methods and analysis provided to allow replication by others?

Partly

Reviewer Expertise:

Clinical trial registration and reporting, meta-research, regulatory science

I confirm that I have read this submission and believe that I have an appropriate level of expertise to confirm that it is of an acceptable scientific standard.
